# The Finnish New Variant of *Chlamydia trachomatis* with a Single Nucleotide Polymorphism in the 23S rRNA Target Escapes Detection by the Aptima Combo 2 Test

**DOI:** 10.3390/microorganisms7080227

**Published:** 2019-07-31

**Authors:** Kati Hokynar, Kaisu Rantakokko-Jalava, Antti Hakanen, Marika Havana, Laura Mannonen, Pia Jokela, Satu Kurkela, Maija Lappalainen, Magnus Unemo, Mirja Puolakkainen

**Affiliations:** 1 Virology, University of Helsinki and Helsinki University Hospital, 00014 Helsinki, Finland; 2 Department of Clinical Microbiology, Turku University Hospital, 20500 Turku, Finland; 3 Department of Virology, University of Turku, 20500 Turku, Finland; 4 SYNLAB Finland, 00310 Helsinki, Finland; 5 World Health Organization (WHO) Collaborating Centre for Gonorrhoea and other Sexually Transmitted Infections (STIs), National Reference Laboratory for STIs, Department of Laboratory Medicine, Faculty of Medicine and Health, Örebro University, SE-701 85 Örebro, Sweden

**Keywords:** *Chlamydia trachomatis*, mutation, nucleic acid amplification test, diagnostics, false negative

## Abstract

In 2019, more than 200 cases of *Chlamydia trachomatis* negative/equivocal by the Aptima Combo 2 assay (AC2, target: 23S rRNA) with slightly elevated relative light units (RLUs), but positive by the Aptima *Chlamydia trachomatis* assay (ACT, target: 16S rRNA) have been detected in Finland To identify the cause of the AC2 CT false-negative specimens, we sequenced parts of the CT 23S rRNA gene in 40 specimens that were AC2 negative/equivocal but ACT positive. A single nucleotide polymorphism (SNP; C1515T in the *C. trachomatis* 23S rRNA gene) was revealed in 39 AC2/ACT discordant specimens. No decrease in the number of mandatorily notified *C. trachomatis* cases was observed nationally in Finland in 2010–2019. When RLUs obtained for AC2 negative specimens were retrospectively evaluated in 2011–2019, a continuous increase in the proportion of samples with RLUs 10–19 was observed since 2014, and a slight increase in the proportion of samples with RLUs 20–84 in 2017–2019, indicating that the Finnish new variant of *C. trachomatis* might have been spreading nationally for several years. This emphasizes that careful surveillance of epidemiology, positivity rate and test performance are mandatory to detect any changes affecting detection of infections.

## 1. Introduction

*Chlamydia trachomatis* is the most common bacterial sexually transmitted infection globally with more than 127 million new cases among adults estimated in 2016 [1]. In 2017, more than 400,000 *C. trachomatis* infections were reported by 26 European countries to the European Centre for Disease Prevention and Control (ECDC) [2]. Sexually active young people have the highest risk of being infected with *C. trachomatis*. The clinical outcome of the infection varies: males most frequently have urethritis and females cervicitis. However, the vast majority of chlamydial infections, particularly in females, are asymptomatic [3]. This poses significant challenges for detection.

Laboratory diagnosis of *C. trachomatis* infection is currently mainly based on nucleic acid amplification tests (NAATs) due to their superior performance characteristics. NAATs enable very sensitive and specific detection of microbial nucleic acid present even in small quantities. No viable infectious microbes are required, so transport and storage conditions of specimens are less critical. In addition, less invasively collected samples, including first void urine (FVU) specimens from males and vaginal swabs from females, are very effective for *C. trachomatis* NAAT testing which facilitates self-sampling and screening of asymptomatic infection.

Microbial sequences selected as detection targets for primers and probes in diagnostic NAATs must be conserved and specific. The genome of *C. trachomatis* consists of chromosomal DNA and a multicopy cryptic plasmid. Most of the US FDA-approved NAATs detecting *C. trachomatis* and *Neisseria gonorrhoeae* are widely used in high-resourced settings and include PCR (Abbott RealTime CT/NG assay, Roche Cobas CT/NG Test, Cepheid GeneXpert Xpert CT/NG, Becton Dickinson Molecular Diagnostics BD Max CT/GC/TV), strand displacement amplification (SDA; BD ProbeTec Viper) and transcription mediated amplification (TMA; Hologic Aptima Combo 2 assay (AC2)) technology. These technologies amplify chlamydial cryptic plasmid DNA, genomic DNA or rRNA, and the Abbott and Roche NAATs have dual targets for *C. trachomatis*. In theory, use of a multicopy target increases sensitivity, and the exploitation of the multicopy cryptic plasmid as well as multicopy ribosomal RNA as NAAT targets for *C. trachomatis* results in high sensitivity [4]. Despite all these advantages, use of NAATs is not without challenges. Microbial, including chlamydial, genomes are not stable, but must evolve to survive through minor mutations, insertions, deletions, and horizontal gene transfer [5]. The *C. trachomatis* genome was initially considered to be highly conserved, however, it has now been shown that recombination in *C. trachomatis* intracellularly is frequent in vitro [6] and in vivo [7]. When evolutionary changes take place in the sequence where NAAT primers and/or probe bind, the test performance can be compromised, and the test can become unreliable and clinically useless [8].

Chlamydial diagnostic NAATs have not been spared from such challenges. In 2006, the number of mandatorily notified *C. trachomatis* cases decreased dramatically in the majority of counties in Sweden, and eventually, the Swedish new variant of *C. trachomatis* (the Swedish nvCt) was described [9]. The Swedish nvCT contained a 377 bp deletion in the cryptic plasmid DNA, excluding the target region for the Abbott and Roche *C. trachomatis* NAATs available at that time. This strain was widely spread nationally in Sweden [10], but with exception of Norway few cases were detected outside Sweden [11,12]. Due to the Swedish nvCT, Abbott and Roche developed the dual target NAATs mentioned above. In February 2019, a few urogenital samples reported negative for *C. trachomatis* by the AC2 (target: 23S rRNA) but with slightly elevated detection signal (relative light units, RLUs), and positive for *C. trachomatis* by Allplex STI test were detected in Turku, Finland [13]. These samples tested positive by the Aptima *C. trachomatis* assay (ACT; target: 16S rRNA) provided by Hologic. Later, several similar cases of *C. trachomatis* that were negative/equivocal by AC2 but positive by ACT were identified in several laboratories using AC2 in Finland. During March–April 2019, 87–100% of specimens with RLUs 20–84 interpreted by the assay software as negative or equivocal and 20% of those with RLUs 15–19 interpreted by the assay software as negative were shown to be positive for *C. trachomatis* when using a test with alternate target (ACT) [13].

We retrospectively analyzed the *C. trachomatis* incidence in Finland (2010–2018), and positivity rates as well as number and proportion of AC2 negative specimens with slightly elevated RLUs (20–84) from 2016–2018 in three laboratories in Finland and negative specimens from 2011–2019 in Clinical Microbiology Laboratory of Helsinki University Hospital (HUSLAB). We also sequenced parts of the 23S rRNA gene and determined the *C. trachomatis* ompA-based genotype in 40 specimens that were AC2 negative/equivocal but ACT positive.

## 2. Materials and Methods

### 2.1. Retrospective Analysis of C. trachomatis Incidence, Positivity Rates and AC2 RLUs

Incidence data of sexually transmitted *C. trachomatis* infection in 2010–2018 from the Infectious Diseases Register (NIDR) maintained by National Institute of Health and Welfare (THL, www.thl.fi) was used. Furthermore, data was collected on the percentage of specimens positive for *C. trachomatis* and on the percentage of negative specimens with RLUs 10–19 and 20–84 in AC2 tests in three major Finnish laboratories; Clinical Microbiology Laboratory of Turku University Hospital (Tyks, serving hospital district of Southwest Finland), Clinical Microbiology Laboratory of Helsinki University Hospital (HUSLAB, serving hospital district of Helsinki and Uusimaa and hospital district of Kymenlaakso), and United Medix Laboratories Ltd (UML, part of SYNLAB Finland, serving private sector clients and Finnish Student Health Service organization operating in the whole of Finland).

### 2.2. Samples

Anonymized samples collected with the Aptima Urine Specimen Collection Kit for Male and Female Urine Specimens or with the Aptima Kit for Endocervical and Male Urethral Swab Specimens were available for this study. Samples were sent for CT/NG testing with AC2 run on automated Panther instruments and the test results were automatically interpreted by the Aptima Assay software [14]. The cutoffs are RLU 25 for equivocal (RLU ≥ 25– < 100) and 100 for a positive result if only a chlamydial signal is detected. If both chlamydial and gonococcal signals are present or the chlamydial result is indeterminate, the cutoff for *C. trachomatis* equivocal is 85 RLU (RLU ≥ 85– < 250). Samples with ≥250 RLU are positive for *C. trachomatis*, if both chlamydial and gonococcal signals are present [14]. Samples that were negative in the AC2 test but positive in ACT were further investigated. Six such specimens from Tyks, 21 from HUSLAB, and 13 from UML were available for this study (the specimens were collected between January and April 2019, except sample No. 6 that was collected in June 2018). For comparison, five AC2 positive first-void urine samples and 26 *C. trachomatis* positive swab specimens collected in Universal Transport media in 2009–2015 for culture were included in the analysis. The study was approved by the Independent Institutional Review Board of Hospital District of Helsinki and Uusimaa (12 June 2019; HUSLAB 88§/2019).

### 2.3. DNA Extraction

Genomic DNA was extracted from all specimens using the Maxwell® 16 Viral Total Nucleic Acid Purification Kit (Promega, Madison WI, USA). Briefly, 300 μL of each specimen was mixed with 300 μL of lysis buffer supplemented with 30 μL proteinase K and incubated at 56 °C for 10 min. DNA was then extracted from the specimen lysate with the Maxwell 16 instrument and the isolate DNA was eluted in 50 μL of nuclease free water.

### 2.4. PCR and Sequencing

Overlapping fragments of the 23S rRNA gene of *C. trachomatis* were amplified in duplicate with PCR. The primers used for PCR and sequencing are shown in Table 1. The PCR reactions of 25 μL contained 12.5 μL Maxima Probe/ROX qPCR Master Mix (2×) (Thermo Scientific, Waltham, MA, USA), and 200 nM of forward and reverse primers (purchased from Integrated DA technologies). Amplification was performed on a 7500 Real-Time PCR system (Applied Biosystems, Foster City, CA, United States). Cycling conditions were 95 °C 10 min initial denaturation, followed by 45 cycles of 95 °C/15 s, 62 °C/15 s and 72 °C/1 min. PCR products were visualized on 1% agarose gel by electrophoresis. The PCR amplicons were then purified (Illustra ExoProStar 1-Step kit, GE Healthcare Life Sciences, Buckinghamshire, UK) and the forward and reverse strands of the PCR products were Sanger sequenced twice in the Institute for Molecular Medicine, Finland. Sequences were compared to the known *C. trachomatis* sequences in the NCBI database by BLAST analysis (megablast) and multiple sequence alignment. Additionally, the previously sequenced Finnish *C. trachomatis* low-passage number isolates [15] from AC2 positive patients (the sequencing data deposited in the European Nucleotide Archive, ENA, http://www.ebi.ac.uk/ena, [7]) were used in sequence comparisons.

### 2.5. Genotyping

The *C. trachomatis* specimens were genotyped using an earlier developed ompA-based PCR method [17] with previously described modifications [11].

## 3. Results

### 3.1. C. trachomatis Incidence

**Figure 1:**
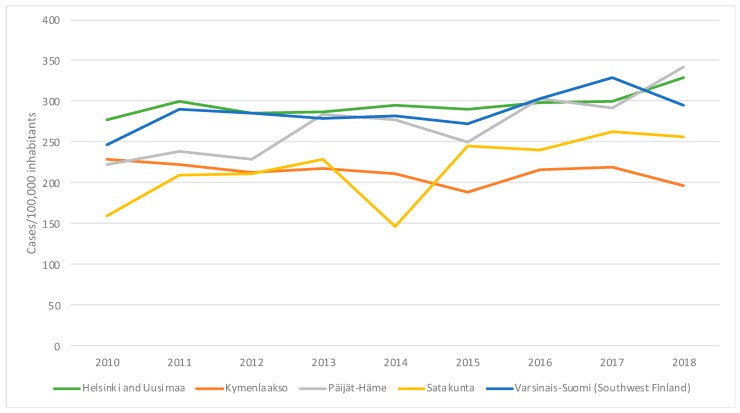
Incidence (cases/100,000 inhabitants) of *Chlamydia trachomatis* in five Finnish hospital districts using the Aptima Combo 2 assay.

The incidence based on the mandatory laboratory notifications of *C. trachomatis* cases in five hospital districts where AC2 is used, from 2010 to 2018, is presented in Figure 1 (data from NIDR). The figure shows about 10% decrease in the incidence of notified *C. trachomatis* cases in the hospital districts of Southwest Finland (Turku area) and Kymenlaakso in 2018. However, these changes did not reach statistical significance. No decrease in the incidence of *C. trachomatis* was observed in 2017–2018 in the hospital districts of Helsinki and Uusimaa, and Päijät-Häme. No decrease in the number of mandatorily notified *C. trachomatis* cases was observed nationally in Finland in 2010–2019.

### 3.2. Retrospective Analysis of C. trachomatis Positivity Rates

**Figure 2:**
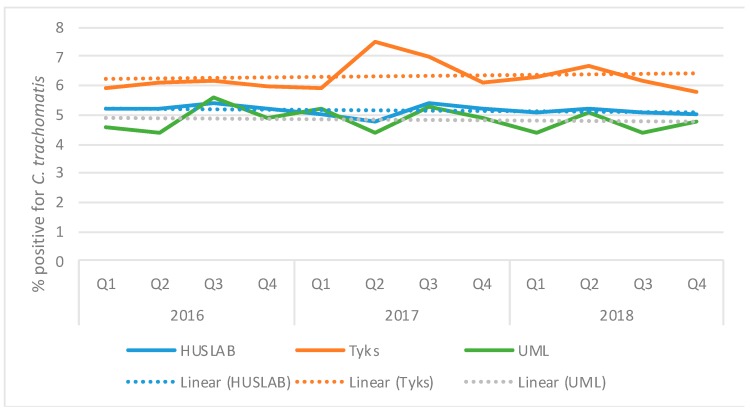
Quarterly proportions of Aptima Combo 2 specimens positive for *C. trachomatis* in three diagnostic laboratories in Finland in 2016–2018. The total number of specimens included was 287,000 (Clinical Microbiology Laboratory of Helsinki University Hospital (HUSLAB), serving hospital district of Helsinki and Uusimaa and hospital district of Kymenlaakso) and 61,000 (Clinical Microbiology Laboratory of Turku University Hospital (Tyks), serving hospital district of Southwest Finland). The number of United Medix Laboratories Ltd (UML) samples was not available.

The percentage of AC2 specimens interpreted by the Panther instruments as positive for *C. trachomatis* in three laboratories is presented in Figure 2. In 2016–2017, approximately 6%, 5%, and 5% of AC2 specimens were positive in Tyks, HUSLAB, and UML, respectively. In Tyks, the percentage of positive samples was exceptionally high in 2017, reflected also as a higher incidence in Figure 1. There was a small but continuous decrease in the positivity rate in Q2–Q4/2018 in Tyks. In HUSLAB, there was a continuous slowly decreasing trend in the proportion of positive samples since 2011, but no major changes during 2016–2018.

### 3.3. Retrospective Analysis of RLUs

**Figure 3:**
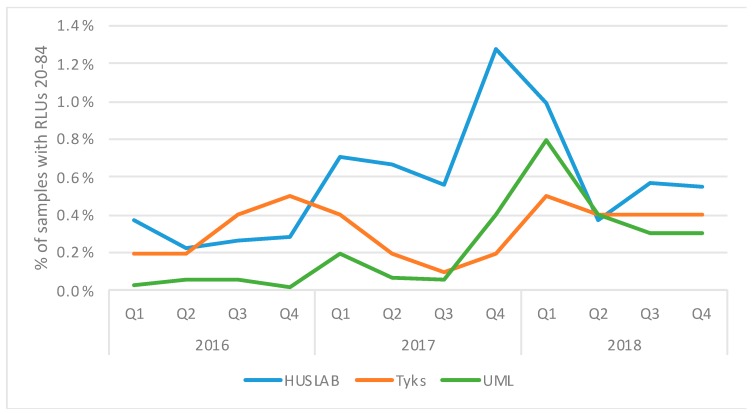
Quarterly proportions of Aptima Combo 2 specimens yielding relative light unit (RLU) 20–84 and flagged negative for *C. trachomatis* in three diagnostic laboratories in Finland in 2016–2018. The total number of specimens included was 212,000 (HUSLAB) and 66,000 (Tyks). The number of UML samples was not available.

During March–April 2019, 87–100% of specimens with RLUs 20–84 interpreted by the Panther system as negative or equivocal were shown to be positive for *C. trachomatis* when using a test with alternate target (ACT) [13]. Figure 3 shows the proportions of AC2 samples with RLU 20–84 in the three laboratories in 2016–2018. In HUSLAB, their proportion started to increase in the first quarter of 2017, and in the last quarter of 2017, their proportion reached 1.2% of all AC2 samples. The maintenance history of the Panther instruments revealed no technical or software issues connected with the increase. At UML, the proportion of samples with RLU 20–84 increased in a similar fashion, but the percentages were lower (reaching 0.8% in Q1/2018). In Tyks, the increase in the percentage of samples with RLUs 20–84 was observed somewhat later, namely in 2018. In Tyks, already in 2016–2017, increased proportions of specimens with elevated RLU were seen, but this was mainly due to technical issues as the proportions decreased after Panther service visits.

When proportioned to the samples reported as positive for *C. trachomatis*, the percentage of samples negative for *C. trachomatis* with RLU 20–84 in HUSLAB was 8.6%, in Tyks 5.7% and in UML 7.1%. This may represent the amount of missed *C. trachomatis* cases, as in 2019, up to 100% of specimens with RLUs 20–84 interpreted by the assay software as negative or equivocal were shown to be positive for *C. trachomatis* when using a test with alternate target (ACT) [13].

**Figure 4:**
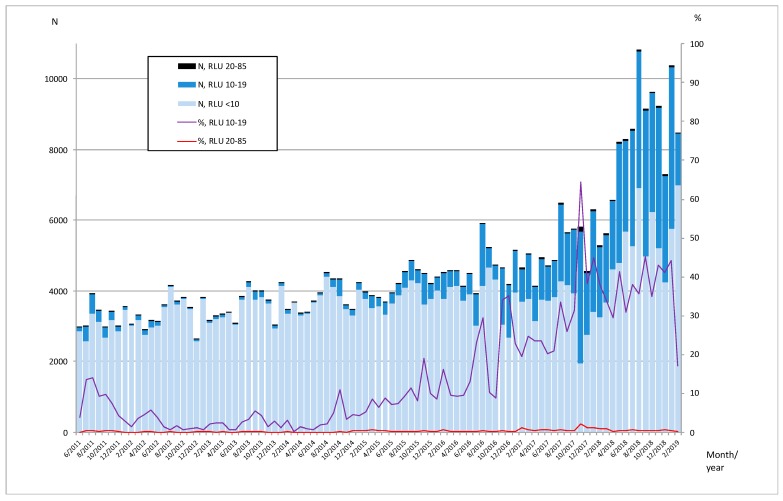
AC2 negative specimens analyzed in the Helsinki University Hospital Laboratory from June 2011 to February 2019 according to their RLU result. Data for this analysis were available from 434,000 specimens. Black bars represent negative specimens yielding an RLU result between 20 and 85; the blue bars represent negative specimens yielding an RLU result between 10 and 19; and the light blue bars represent all other negative specimens (RLU < 10). The red line represents the proportion (%) of specimens yielding an RLU result between 20 and 85, and the purple line represents the proportion (%) of specimens yielding an RLU result between 10 and 19.

Retrospective analysis of 433,873 AC2 negative samples tested in HUSLAB from June 2011 to February 2019 (Figure 4) indicates that the proportion of specimens with RLUs 10–19 started to increase in late 2014 (10%) and reached 40% of negatives in 2018 in that area. This might be due to technical issues (related to AC2 reagents or Panther instruments) or the appearance and gradual dissemination of a *C. trachomatis* variant giving slightly elevated RLUs in the AC2 test.

### 3.4. PCR and Sequencing of 23S rRNA Gene

**Figure 5:**
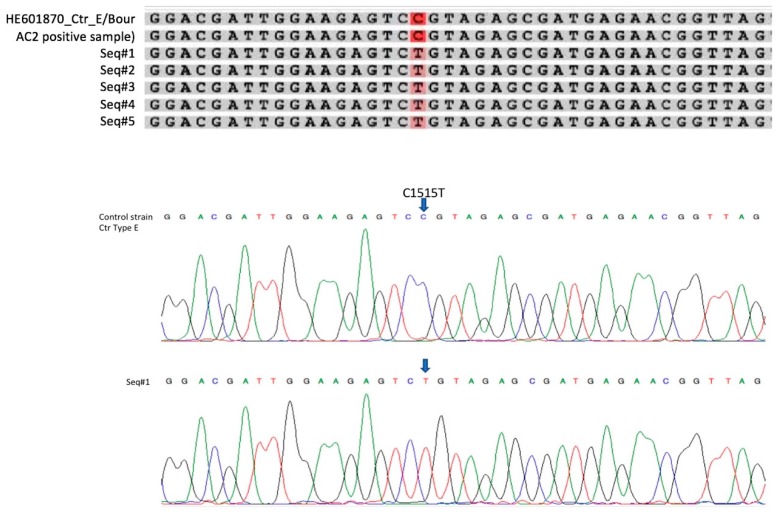
The single mutation C1515T in 23S rRNA gene of *Chlamydia trachomatis*. Alignment of sequences of samples 1–5 (Table 2), an Aptima Combo 2 positive sample and a reference strain, and chromatograms of control strain genotype E and sample 1 showing the mutation.

Initially, five primer pairs were used to amplify *C. trachomatis* sequences coding for 23S rRNA of 11 specimens (specimens 1–5 and 7–12 in Table 2). All fragments were successfully amplified and sequenced in six, four fragments in three, and three fragments in two specimens. Sanger sequencing detected a single nucleotide polymorphism (SNP) at position C1515T in the *C. trachomatis* 23S rRNA gene in all AC2 negative but ACT positive samples when compared to the *C. trachomatis* E/Bour (HE601870) (Figure 5) sequence. The SNP was verified in both the forward and reverse strands of the sequenced V2 and V3 PCR products, with high quality values (Q-value > 30 by FinchTV, Geospiza). This SNP verifies that AC2 negative but ACT positive specimens contained the Finnish new variant of *C. trachomatis* (FI-nvCT) [18]. As this was the only mutation detected in the sequenced regions of the 23S rRNA gene, only primer pair V2 was used to amplify 29 additional AC2 negative/equivocal and ACT positive specimens, and the five AC2 positive specimens. Twenty-eight of these 29 AC2 negative/ACT positive specimens had the C1515T SNP, whereas one AC2 equivocal specimen (number 16) and the five AC2 positive specimens did not have this mutation. The equivocal specimen yielded RLU 66 in AC2 and RLU 6948 in ACT. As the specimen was tested with ACT and then included in this study, the AC2 test could not be repeated.

DNA sequences from 32 Finnish low-passage-number cervical isolates [15], including 12 representing genotype E, from an earlier study [7] were also analyzed. None of these isolates collected from AC2 positive patients in Finland in 2009–2011 had this SNP. Twenty-six culture-positive swabs from 2009–2015 collected in Helsinki were also analyzed for the presence of this SNP, and they all contained a 23S rRNA gene of wild-type.

### 3.5. Genotyping

All specimens that were AC2 negative but ACT positive contained *C. trachomatis* genotype E. The AC2 equivocal specimen (number 16) with wild-type 23S rRNA sequence contained *C. trachomatis* genotype G (Table 2).

## 4. Discussion

The SNP C1515T in the 23S rRNA gene was the only difference initially observed between 10 AC2 negative or equivocal/ACT positive and AC2 positive samples [13]. Although the samples containing this SNP (FI-nvCT samples) had RLUs slightly above the background level (RLU 15–46), they were initially interpreted negative for *C. trachomatis* by the Panther instruments. In the present study, the presence of the FI-nvCT was further confirmed in an additional 29 AC2/ACT discordant samples. The ACT has higher analytical sensitivity than AC2, which may explain why AC2 negative/equivocal but ACT positive test results are not necessarily related to the FI-nvCT (see sample No. 16 in Table 2). However, 17 of the 20 AC2 negative/ACT positive samples with RLUs 15-24 were positive for *C. trachomatis* by a multiplex PCR (Allplex™ CT/NG/MG/TV, Seegene) test in HUSLAB. This implies that the majority of our discordant results were not due to a low bacterial load. All FI-nvCT samples that could be genotyped were *C. trachomatis* type E. As genotype E is the most common genotype in Finland [11] and in most other countries globally [7], this observation neither confirms nor excludes that the variant strain is of clonal origin. Whole genome sequencing of the FI-nvCT specimens will disclose whether there are additional changes in the bacterial genome. Notably, the Swedish nvCT strain was also of genotype E, and, in addition to the 377 bp deletion, it had a 44 bp duplication in the plasmid DNA [19].

All three methodological steps in the AC2 test could theoretically be affected by a mutation: target capture-based sample preparation, transcription mediated amplification of rRNA through DNA intermediates, and detection of amplified RNA using hybridization protection assay (HPA). As soon as the initial false-negative AC2 CT specimens were detected [13], the manufacturer was informed and after internal investigation, the manufacturer disclosed that 23S rRNA gene C1515T SNP we discovered yields a significantly suppressed *C. trachomatis* detection probe-signal [20]. In HPA, chemiluminescent acridium ester-labeled probes bind to complementary RNA amplicons. During detection, alkaline hydrolysis destroys chemiluminescence of unhybridized probes, whereas in hybridized probes, the acridium ester label is sterically protected from hydrolysis. HPA has earlier been shown to detect SNPs in synthetic DNA and RNA oligos, and SNPs were shown to reduce the signal to ≤1% of the signal obtained with a 100% matched target sequence [21]. This is in accordance with our observation: samples with the FI-nvCT resulted in very significantly reduced signal in the AC2 and were consequently interpreted as negative. To streamline clinical sample testing with Aptima, Hologic is developing a new assay that specifically targets the FI-nvCT [20,22]. Recently, initial evaluation revealed that two cases of FI-nvCT have been identified in Sweden [18]. As ECDC has been notified and ECDC has informed public health officials in Europe, we expect more information on the epidemiology of FI-nvCT to accumulate.

At present, it remains unknown when the FI-nvCT appeared in Finland. The earliest available specimen that contained the FI-nvCT was sampled in June 2018 in Turku. The patient’s FVU sample had been AC2 negative for *C. trachomatis*, and a week later a new sample was referred to be tested with the Anyplex II STI-5 multiplex PCR and stored. Anyplex II STI-5 targeting several genital mycoplasmas and *Trichomonas vaginalis* but not *C. trachomatis*, was originally introduced in Turku in 2014 as a secondary STI-test mainly to detect *Mycoplasma genitalium* in symptomatic patients negative for *C. trachomatis* and *N. gonorrhoeae*. Had that panel originally contained *C. trachomatis* we might have caught the variant 8 months earlier, showing that the use of a backup NAAT may be an effective means to catch possible diagnostic escape mutants when primary testing remains negative in symptomatic patients. Now, the first FI-nvCT cases in February 2019 were discovered by chance and without testing for *C. trachomatis* with a NAAT using a different target (Allplex STI Essential) they would have remained unnoticed. Retrospectively analyzed RLUs of specimens from 2016–2018 showed that the proportion of AC2 negative specimens with RLUs 20–84 started to increase in 2017. Additionally, the analysis of nearly 434,000 AC2 negative samples tested in HUSLAB indicates that the proportion of samples with RLUs 10–19 started to increase already in 2014. There are a number of explanations for such a phenomenon, including those related to slight variations in the reagent lots and elevated RLUs associated with incompletely dissolved reagents, as well as technical issues of Panther instruments possibly causing a gradual shift in the RLU background level. Another explanation is the appearance of FI-nvCT in the Helsinki metropolitan area in late 2014, and gradually spreading thereafter. Interestingly, among patients confirmed to carry the FI-nvCT, one (No. 12) had given a sample with RLU 23 already in 2015. Further epidemiological and molecular investigations are needed to establish the time of appearance of the FI-nvCT in Finland.

It is noteworthy that, contrary to the episode in Sweden with the appearance of the Swedish nvCT, no decrease in the number of mandatorily notified *C. trachomatis* cases was observed nationally in Finland in 2010–2019. Locally, however, the incidence of *C. trachomatis* cases showed a decreasing tendency in the hospital district of Southwest Finland (Turku) in 2018, where AC2 is used (the change did not reach statistical significance). Most *C. trachomatis* cases in Finland are reported from the hospital district of Helsinki and Uusimaa, and although a majority of samples are being diagnosed by AC2, the incidence has been on the increase since 2015 suggesting that the clearly rising incidence of *C. trachomatis* infections is masking the presence of FI-nvCT in that area. In HUSLAB, Tyks and UML, the proportion of FI-nvCT is estimated as 6–9% of *C. trachomatis* cases.

The *C. trachomatis* rRNA operon contains the genes encoding the chlamydial 5S, 16S, and 23S rRNA and the C1515T SNP found here is in the region coding for domain II of *C. trachomatis* 23S rRNA. There are two copies of the rRNA operon in the *C. trachomatis* genome [23] and the 23S rRNA gene in the two operons has been considered to be identical. Whether the observed mutation is present in one copy or both copies of the operon is not known yet. However, according to the Sanger sequencing chromatograms there are no mixed sequences which indicate that the C1551T is likely in both copies (also supported by our preliminary PCR data). Nevertheless, whole genome sequencing, which is in progress, will be needed to provide clearer evidence regarding this issue. 16S rRNA and 23S rRNA are structural components of ribosomes and their function as well as structure is well preserved. SNPs in the 23S rRNA peptidyltransferase region within domain V are associated with resistance to macrolides in other bacteria, and domain II can interact with domain V [24]. Based on the position of the C1515T mutation in 23S rRNA, most likely it does not affect susceptibility to macrolides, however, it cannot be completely excluded. It remains to be studied, whether the FI-nvCT has altered biological properties such as altered transmissibility, pathogenicity, or general fitness.

Variants of microbes evolve, and their emergence can create detection problems with NAATs [8]. Even SNPs can severely compromise nucleic acid amplification and/or detection when unfavorably positioned, as was observed in this study. When evaluating and implementing diagnostic tests, known variants can be taken into consideration while an emerging diagnostic escape variant is more problematic for clinical laboratories, since established quality assessment programs cover only known strains of microbes. NAATs with dual targets or frequent testing of random samples of negatives with another assay having another target would be helpful. In addition, careful and preferably automated monitoring of long-term trends (epidemiology, positivity rate, and assay signal values) is an essential component of quality confirmation in diagnostics.

## Figures and Tables

**Table 1:** The primers used in this study.

Name	Sequence	Amplicon Size	Reference
U23 23SIG	F: 5′-GATGCCTTGGCATTGATAGGGGATGAAGGA-3′ R: 5′-TGGCTCATCATGCAAAAGGCA-3′	604 bp	[16]
23S V1	F: 5′-AAGACCGACCTCAACACCTG-3′	823 bp	This study
	R: 5′-GCGATGTCGGTTTTATGCTT-3′		
23S V2	F: 5′-GGCTTACCAACGGAAATCAA-3′	741 bp	This study
	R: 5′-GCGATGTCGGTTTTATGCTT-3′		
23S V3	F: 5′-AGCGAAGGAATGACGGAGTA-3′	656 bp	This study
	R: 5′-AAGGTTCACGGGGTCTTTTC-3′		
23S V4	F: 5′-TGAACCTAAGCCCTGGTGAATGGC-3′	897 bp	This study
	R: 5′-CTTGAGGGAGGCTTGGCATT-3′		

**Table 2:** Characteristics of specimens with negative/equivocal * Aptima Combo 2 and positive Aptima *Chlamydia trachomatis* results.

Specimen Number, GenBank Accession Number	Specimen Type	Patient’s		AC2 RLU (×1000)	ACT RLU (×1000)	Genotype MOMP	Sequence of Amplicons				
		Sex	Age				U23, 23 SIG	V1	V2	V3	V4
1 MN006991	FVU	M	32	26	6405	E	WT	WT	C1515T	C1515T	WT
2	FVU	M	37	28	6395	E	WT	WT	C1515T	C1515T	WT
3	FVU	F	35	21	6679	E	NA	WT	C1515T	C1515T	NA
4	FVU	F	18	46	6118	E	WT	WT	C1515T	C1515T	WT
5	FVU	F	42	20	6434	E	WT	WT	C1515T	NA	WT
6	FVU	M	28	19	6850				C1515T		
7	FVU	F	20	39	7137	E	WT	WT	C1515T	C1515T	WT
8	FVU	F	23	42	7264	E	WT	WT	C1515T	C1515T	WT
9	FVU	M	26	20	7337	E	WT	WT	C1515T	C1515T	WT
10	FVU	F	24	25	6827	E	NA	WT	C1515T	C1515T	NA
11	FVU	M	27	26	7318	E	WT	WT	C1515T	NA	WT
12	FVU	M	26	26	7250	E	WT	WT	C1515T	C1515T	NA
13	FVU	M	37	28	7221	E			C1515T		
14	FVU	F	18	26	7163	E			C1515T		
15	FVU	F	21	27	6991	E			C1515T		
16	FVU	F	20	66 *	6948	G			WT		
17	Pharyngeal swab	F	24	21	7506	E			C1515T		
18	FVU	M	29	23	7102	E			C1515T		
19	FVU	M	24	24	7324	E			C1515T		
20	FVU	F	24	24	7066	E			C1515T		
21	FVU	F	29	25	7083	E			C1515T		
22	FVU	F	22	25	7063	E			C1515T		
23	Cervical swab	F	26	28	7135	E			C1515T		
24	FVU	M	21	17	6882	E			C1515T		
25	Rectal swab	F	18	15	6920	NA			C1515T		
26	Cervical swab	F	28	18	7008	E			C1515T		
27	Pharyngeal swab	M	30	23	6485	E			C1515T		
28	FVU	F	34	22	7121	E			C1515T		
29	FVU	F	38	28	7142	E			C1515T		
30	FVU	M	50	24	6803	E			C1515T		
31	FVU	F	24	20	6748	E			C1515T		
32	FVU	F	26	32	6882	E			C1515T		
33	FVU	M	33	25	6848	E			C1515T		
34	FVU	M	32	25	7087	E			C1515T		
35	FVU	M	23	33	6870	E			C1515T		
36	FVU	F	33	18	6899	E			C1515T		
37	FVU	F	29	23	6944	E			C1515T		
38	FVU	M	38	29	6826	E			C1515T		
39	FVU	M	34	27	6673	E			C1515T		
40	FVU	M	29	26	6806	E			C1515T		

*—equivocal; NA—not available (sequence not readable); WT—wild type; C1515T—mutation in position 1515.
